# Supplementary material for: A general method for the creation of dilational surfaces
Source: Nat Commun. 2019 Nov 15;10:5180. doi: 10.1038/s41467-019-13134-0 (PMC6858453; doi:10.1038/s41467-019-13134-0)
Supplement: Supplementary file 1 — Description of Additional Supplementary Files [file 41467_2019_13134_MOESM1_ESM.docx]

Description of Additional Supplementary Files

**Supplementary Video 1** (Octahedron.mp4): A movie of a dilational octahedron that was obtained using our method, moving with its range of motion. The structure in this movie corresponds to the first example in this paper.

**Supplementary Video 2** (Cardioid.mp4) A movie of a dilational cardioid that was obtained using our method, moving with its range of motion. The structure in this movie corresponds to the second example in this paper.

**Supplementary Video 3** (StanfordBunny.mp4) A movie of a dilational Stanford bunny that was obtained using our method, moving with its range of motion. The structure in this movie corresponds to the third example in this paper.
