# Supplementary material for: A general method for the creation of dilational surfaces
Source: Nat Commun. 2019 Nov 15;10:5180. doi: 10.1038/s41467-019-13134-0 (PMC6858453; doi:10.1038/s41467-019-13134-0)
Supplement: Supplementary file 2 — Supplementary Information [file 41467_2019_13134_MOESM2_ESM.pdf]

Supplementary Information
to
A General Method for the Creation of Dilational Surfaces

Freek G.J. Broeren[1 * †] , Werner W.P.J. van de Sande[1 *], Volkert van der Wijk[1], and Just L. Herder[1]

[1]Department of Precision and Microsystems Engineering, Delft University of Technology, Mekelweg 2, 2628 CD Delft, the Netherlands
[*]These authors contributed equally to this work
[†]Corresponding author: f.g.j.broeren@tudelft.nl

Supplementary Videos

We include videos of the dilational octahedron, cardioid and Stanford bunny as they move within their range of motion. These movies correspond to the examples presented in the paper.

Supplementary Data

The input STLs, directed graphs and resulting dilational structures for each of the three examples have been uploaded to the 4TU.ResearchData repository: DOI:10.4121/uuid:36cfec67-aa04-469f-ac13-64e7d95a0c18.

Supplementary Note 1: Graph theory proof

When placing pantograph tiles on a surface, it can happen that the range of motion is severely limited by collisions between neighboring pantograph mechanisms. In order to avoid this problem, the pantograph mechanisms can be placed such that there is always a motion path allowing each of the pantograph mechanisms to move along with its neighbors, thereby avoiding collisions.

In this section we will prove that there always exists such a placement of the pantograph tiles. For this, we represent the triangulated surface as a graph, with one vertex per triangular face and a connection between vertices if they share an edge. If there are holes in the surface, they also get one vertex and are connected to each of the triangular faces neighboring this hole.

In this graph, we represent the motions of the pantograph tiles by directions of the edges. The pantograph tiles either move outside of their described triangle along two edges and into the describe triangle at the other edge or vice-versa.

Using this representation, a motion without collisions between neighboring faces is one where each of the vertices with degree 3 has at least one edge moving into the vertex and one out of the vertex, we call such a vertex *correct*.

Definition 1. *A node v is* correct *if*

$$deg^+(v) > 0 \text{ and } deg^-(v) > 0 \tag{1}$$

In the following, will prove that for every connected graph where each vertex has a degree of at least 2, there exists a *correct orientation*; an orientation for which every node is correct.

First, we observe that if we have a correct directed graph, we can always add extra edges and orient them such that the composite graph is also correct.

Lemma 1. *Let G be a connected graph and H a subgraph of G with the same set of nodes. If there exists a correct orientation of H, than there also exists one for G.*

Proof. Choose a correct orientation of the edges of H. To obtain an orientation of G, we only add directed edges to this orientation. In doing this, the in- and outdegree of the nodes of G can only increase, therefore, this orientation will always be correct. □

Next, we observe that the union of two graphs that each have a correct orientation must also have a correct orientation.

Lemma 2. *Let G be a simple graph, and let H_1 and H_2 be subgraphs of G. Let H be the union of H_1 and H_2. If H_1 and H_2 each have a correct orientation, then H also has one.*

Proof. Choose correct orientations of both H_1 and H_2. Now, define an orientation on H according to the following: If e is an edge of H that is present in H_1, direct it according to the orientation of H_1. If e is present in H_2, but not in H_1, we orient is according to the orientation of H_2. We claim that the orientation that is built up in this way is correct.

Let v be a node in H. If v is in H_1, then the $\deg^+(v)$ and $\deg^-(v)$ in H are at least those it has in H_1, so v is a correct node in H. If v is in H_2, but not in H_1, it will have an in- and outdegree at least equal to those in H_2, so it is also a correct node. This covers all nodes in H and therefore the orientation built up according to the above procedure is correct. □

From the above two lemmata, it follows that the existence of a correct orientation of a graph is a local property:

Lemma 3. *Let G be a finite simple graph. There exists a correct orientation for G if and only if for each vertex v in G, there exists a subgraph H of G that contains v and has a correct orientation.*

Proof. If there exists a correct orientation of G, we can choose $H = G$. For the converse implication, choose a subgraph H_v for each vertex v in G, that has a correct orientation. Let H be the union of all subgraphs H_v. If we repeatedly apply Lemma 2, we find that there must exist a correct orientation of H. Furthermore, since H contains all nodes of G, G must have a correct orientation by Lemma 1. □

Finally, to prove that for any connected graph with vertices of at least degree 2 there exists a correct orientation, we only need to prove that every node of such a graph lies on a subgraph that has a correct orientation:

Lemma 4. *Let G be a simple graph in which every node has at least degree 2. Then, G has a correct orientation.*

Proof. Let v be a random node in G. Following the above lemmata and proposition, we only need to prove that v lies in a subgraph H of G that has a correct orientation.

If v lies on a cyclic subgraph of G, then we choose H to be this cycle and orient the edges along the cycle, creating a correct orientation.

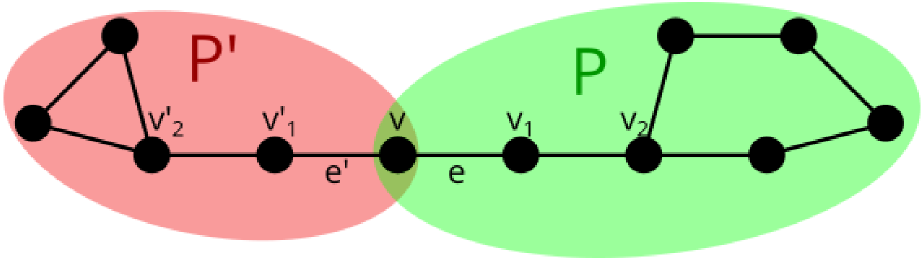

Supplementary Figure 1: We construct a subgraph that has a correct orientation containing v by following the edges from v to create paths P and P'. The resulting graph can be oriented such that every node has a nonzero in- and outdegree.

If v does not lie on a cyclic subgraph of G, we construct H as follows. Choose two edges e, e', connected to v. We construct a path P in G by starting at v, following e to the next node v_1, then following another edge connected to v_1 to a node v_1 and so on. We stop when we encounter a node that has already been visited. The path P now looks like a lasso or a capital letter P. We can do this because every node has at least degree 2, so we can always continue constructing this path. Similarly, we construct a path P' by starting at v and now walking along e' to a node v_1' and so on untill we encounter a node that is already in P'. Such a resulting graph is shown in Supplementary Fig. 1. Note that the only node in both P and P' is v, because otherwise v would have been part of a cyclic subgraph. The resulting graph H always has the shape of an elongated figure 8. Such a graph always has a correct orientation. Since this can be done for every node v in G, we have proven that G has a correct orientation. □

With this, we have proven that for every connected graph there exists a correct orientation and therefore that for every triangulated surface there exists a placement of pantograph tiles such that there is a motion path with no collisions between neighboring faces.

Supplementary Note 2: Tiling Algorithm

To create the tiled structures presented in this paper, an algorithm was used to determine correct tilings for the given surfaces. The algorithm we used is based on flows through the dual graph to the surface.

We start with a simple undirected graph $G = \{V, E\}$ and we are looking for an orientation $H = \{V, F\}$ which is correct, meaning that every node is correct according to the definition 1. For this, we note that nodes of degree 3 are correct when they are not a source or a sink of the simulated flow. The algorithm then works as follows:

- Create a DiGraph H with no edges and the same vertices as G
- As long as there are vertices in G:
  - Randomly choose an edge e, connecting vertices $v1$, $v2$ in G and remove it from G
  - Simulate a flow from $v1$ to $v2$ through G using a push-relable maximum flow algorithm (preflow-push from the python NetworkX module[1])
  - For all edges through which the flow goes, add them to H in the direction of the flow
  - Add an edge from $v2$ to $v1$ to H
  - If a vertex in H has the same degree as the corresponding vertex in G, remove it from G
- If there are sources and/or sinks of degree 3 in H:
  - If a source s has a neighbor n with degree 3 and indegree > 1:
    - Reverse the direction of the edge between s and n
    - Stop evaluating s
  - If a sink s has a neighbor n with degree 3 and outdegree > 1:
    - Reverse the direction of the edge between s an n
    - Stop evaluating s
  - If a source/sink s has a neighbor n with degree < 2:
    - Reverse the direction of the edge between s and n
    - Stop evaluating s
  - If, for any source or sink, no suitable neighbor could be found:
    - Raise an error

If an error is raised in the third step of the algorithm, the resulting DiGraph H has left-over sources and sinks and therefore does not meet the requirements on the correctness of the vertices. In case, the algorithm is re-run until a working placement is found.

Vertices	10	50	100	200	500	750	1000
Avg. tries per graph	1.000	1.007	1.022	1.043	1.186	1.333	1.552

Supplementary Table 1: We tested our graph direction algorithm on graphs of different sizes, generating 1000 graphs for each size and recording the average number of tries the algorithm needed to find a suitable orientation. The algorithm was successful for all graph sizes, but the number of tries needed increased with graph size.

The flow-based approach ensures that in each iteration of the second step the vertices have at least one edge moving into it and one moving out, since the flow needs to go through each vertex except for the defined source and sink. The reason that sometimes the second step of the algorithm does leave sources and sinks in the graph (that is, vertices with only edges moving out of or into it respectively) is that in general multiple flows need to be simulated to cover the whole graph and that no correlation between the existing directions and the newly simulated flow is incorporated.

To validate this algorithm, we tested it on randomly generated 3-regular graphs (random_regular_graph() from the python NetworkX module[1]). For graphs of various sizes, we recorded how many tries the algorithm needed to find a suitable orientation. For each size, we tested 1000 randomly generated graphs. The results are summarized in Table 1. For all 7000 generated graphs, the algorithm was able to find a suitable orientation.

References

[1] Hagberg, A. A., Schult, D. A. & Swart, P. J. Exploring Network Structure, Dynamics, and Function using NetworkX. In Varoquaux, G., Vaught, T. & Millman, J. (eds.) *Proc. 7th Python Sci. Conf.*, 11–15 (Pasadena, CA USA, 2008).
